# Syndromic surveillance for West Nile virus using raptors in rehabilitation

**DOI:** 10.1186/s12917-017-1292-0

**Published:** 2017-11-29

**Authors:** Alba Ana, M. Perez Andrés, Ponder Julia, Puig Pedro, Wünschmann Arno, Vander Waal Kimberly, Alvarez Julio, Willette Michelle

**Affiliations:** 1 University of Minnesota, St. Paul, MN USA; 2 Universitat Autònoma de Barcelona, Cerdanyola del Vallès, Barcelona, Spain; 3 Univ of Minnesota College of Veterinary Medicine, 1920 Fitch Avenue, St. Paul, MN 55108 USA

**Keywords:** Wildlife rehabilitation, Syndromic surveillance, Raptors, Big data, Time series, West Nile

## Abstract

**Background:**

Wildlife rehabilitation centers routinely gather health-related data from diverse species. Their capability to signal the occurrence of emerging pathogens and improve traditional surveillance remains largely unexplored. This paper assessed the utility for syndromic surveillance of raptors admitted to The Raptor Center (TRC) to signal circulation of West Nile Virus (WNV) in Minnesota between 1990 and 2014. An exhaustive descriptive analysis using grouping time series structures and models of interrupted times series was conducted for indicator subsets.

**Results:**

A total of 13,080 raptors were monitored. The most representative species were red-tailed hawks, great horned owls, Cooper’s hawks, American kestrels and bald eagles. Results indicated that temporal patterns of accessions at the TRC changed distinctively after the incursion of WNV in 2002. The frequency of hawks showing WNV-like signs increased almost 3 times during July and August, suggesting that monitoring of hawks admitted to TRC with WNV-like signs could serve as an indicator of WNV circulation. These findings were also supported by the results of laboratory diagnosis.

**Conclusions:**

This study demonstrates that monitoring of data routinely collected by wildlife rehabilitation centers has the potential to signal the spread of pathogens that may affect wild, domestic animals and humans, thus supporting the early detection of disease incursions in a region and monitoring of disease trends. Ultimately, data collected in rehabilitation centers may provide insights to efficiently allocate financial and human resources on disease prevention and surveillance.

## Background

Wild animals play a key role in the transmission of many infectious diseases into humans by serving as reservoirs for important pathogens such as West Nile virus (WNV), avian influenza virus (AIV), and Lyme disease. Animal health surveillance may contribute to the early detection and prevention of disease outbreaks in human populations [1–3]. In addition to human health, the industries involved in livestock, poultry and fishery production are also vulnerable to infectious disease transmitted by wildlife, which may result in economic losses from disease outbreaks and the potential imposition of stringent trade restrictions [4]. Although there are systems in place for monitoring infectious diseases in humans and some domestic animals, as well as some programs for specific diseases in free-ranging wildlife, there is currently no comprehensive, integrated strategy for monitoring wildlife health issues in the United States [5]. Furthermore, although the need for such a monitoring system has been identified, challenges such as cost, time, case acquisition, and practicality of sampling strategies remain difficult to overcome. Sample collection is currently limited to expensive active surveillance activities, often requiring trapping of animals, or convenience or passive sampling of hunted animals or wildlife submitted to public health or wildlife agencies [6].

Novel approaches, such as syndromic surveillance based on the monitoring of non-specific digital data, may help to enhance current surveillance systems. The Centers for Disease Control have defined syndromic surveillance as an investigational approach based on using real-time health-related data that precedes diagnosis to detect an outbreak of a disease and decrease morbidity and mortality [7]. Besides enhancing the early detection of infectious disease, syndromic surveillance may also contribute to monitoring endemic diseases and/or be used to accumulate proof of absence of a disease [8].

It is estimated that more than 500,000 amphibians, reptiles, birds, and marine and terrestrial mammals are admitted into wildlife rehabilitators across the United States annually [5], representing a diverse array of animal species from disparate geographic regions and a range of ecosystems. Use of wildlife rehabilitation centers as an alternative means of monitoring wildlife and environmental health has been proposed [5, 6, 9–13]. However, there are a number of challenges to the routine use of rehabilitation center data for syndromic surveillance, including: absence of specific surveillance goals and objectives; lack of comprehensive, integrated database systems; limited infrastructure for wildlife rehabilitators; and data quality-, integrity-, and timeline-related issues. Prerequisite for overcoming these challenges and, ultimately, facilitating early detection and prevention of disease incursions, is the implementation of novel surveillance strategies and analytical tools into data routinely collected by wildlife rehabilitation centers. Indeed, it is critical to develop and rigorously validate analytical approaches for monitoring rehabilitation data, including for the detection of aberrations in the data that may indicate a health event. Here, we use the West Nile Virus (WNV) in the state of Minnesota (MN), USA, as a proof-of-concept as to whether the initial incursion of WNV into the state would result in aberrations in raptor rehabilitation data that would be detectable via syndromic surveillance.

West Nile Virus (family *Flaviviridae*, genus *Flavivirus*) is primarily maintained through a bird-mosquito-bird transmission cycle, which sporadically results in epidemics affecting humans and horses. This pathogen has caused significant morbidity and mortality in humans, horses, and wildlife since its introduction into North America in 1999 [14]. The first report of WNV in MN was in two dead crows (*Corvus brachyrhynchos*) in the Minneapolis/St. Paul metropolitan area in July, 2002 [15]; WNV is now endemic/enzootic in MN, manifesting a seasonal pattern during the period of adult mosquito activity. Many species of raptors are susceptible to WNV, which results in a broad range of clinical signs and/or death. It has been suggested that birds represent the first wave of infections during the transmission season, meaning that they may be infected earlier as compared to mosquito pools, humans, or equine cases [6, 16]. This suggests that surveillance of raptors may provide indicators for early detection of WNV incursions into free regions. The study was aimed at assessing the utility of wildlife rehabilitation data to support early detection and monitoring of wildlife pathogen activity as it relates to public, food animal, and environmental health. Specifically, retrospective data from raptors admitted to The Raptor Center (TRC) from Minnesota (MN) were assessed to evaluate its potential for detecting and monitoring of WNV circulation.

## Methods

### Data

This study included retrospective data from raptors admitted to TRC in MN between 1990 and 2014. As a veterinary facility admitting over 800 sick and injured raptors each year, TRC has extensive medical records with the majority of admission data available in a digital format, including clinical signs at admission. Data were collected in Microsoft Access (Microsoft Corp, Redmond, WA) using a relational database. The final data set contained the attributes of “case number”, “species”, “avian group”, “date of admission”, “state” where the bird was found, “age” and “clinical signs”.

### Exploratory descriptive analysis

A descriptive analysis was performed to check data quality with the veterinary clinicians and determine basic traits of the raptor admissions received from MN between 1990 and 2014. Raptor admissions were aggregated by year and month of admission and stratified by avian group, species, age, sex, clinical signs, and state where the birds were found. Raptors could be categorized by age since most species were accurately aged as either a first year (hatch year) or as an adults due to the change in plumage. Frequency of clinical signs was also described for each avian group. Thirty standardized clinical signs recorded upon admission during the entire span of the study were used. Clinical signs were grouped by organ systems into 10 categories, namely: integumentary system (damaged feather/cere/ft, bumblefoot, soft tissue injury); musculoskeletal system (fracture, luxation/subluxation, posterior paralysis); nervous system (convulsions, head tremors, head tilt/disorientation, postural problems/imbalance); gastrointestinal system (anorexia, diarrhea, lesions in mouth, regurgitation); respiratory system (respiratory distress, swollen sinus); urinary system (polydipsia, polyuria, urates in cloaca); special senses (ear injury and ocular disease); non-specific (assymetrical wing beats, injured but alert/feisty, unable to fly or stand, moderate weight loss, not emaciated); systemic (dehydration, weight loss, emaciation, depression/weakness); no problems observed.

### Time series analysis

#### Descriptive analysis of time series for underlying patterns of raptor admissions

The purpose of analysing grouped time series was to identify subsets of raptor admissions that may suggest WNV circulation over time. Frequencies of admissions aggregated by month were assessed by avian taxonomic group, species, clinical signs, and age using grouping time series structures [17–19]. Series were described and compared to assess differences before and after the incursion of WNV in terms of their seasonality and trend. Potential indicators of WNV were selected based on: 1) visual evidence, 2) testing significant changes between the frequencies observed before and after 2002 using non-parametric Mann-Whitney tests [20], and 3) manifestation of WNV-like clinical signs according to previous scientific findings [21, 22].

#### Interrupted time series analysis in the subset of raptors identified as indicator for West Nile virus before and after 2002

Changes over time that could signal WNV circulation were assessed with an interrupted time series (ITS) analysis using the subset of raptors selected as indicator for its circulation [23, 24]. The time series *Y*_*t*_ , that represented the number of raptor admissions by month, was segmented in two parts, namely, data collected between January 1990 and December 2001, and data collected between January 2002 and December 2014. The time series was fitted using a linear regression model including trigonometric covariants as seasonal and cyclical components, such as α_i_ cos (ω_i_t) or/and β_i_ sin (ω_i_t), where *ω*_*i*_ = 2*π*/*T*_*i*_, and *T*_*i*_ corresponded to the periods. Linear-trend components, which slopes are denoted as δ and δ^′^, were also included, and the errors of the linear model (∈_t_) were fitted as autoregressive–moving-average (ARMA) time series with different variances for the two parts considered. The regression coefficients before and after WNV epidemics were estimated introducing a dummy variable (I_t_) , being 1 if t ≤ December 2001 and 0 if t > January 2002. Therefore, the model was expressed as:

$$ {\mathrm{Y}}_{\mathrm{t}}=\left[\left(\mu +\updelta t+\alpha \cos \left(\omega t\right)+\beta \sin \left(\omega t\right)+\cdots +\right){\mathrm{I}}_{\mathrm{t}}\right]+ $$

$$ +\left[\left({\mu}^{\hbox{'}}+{\updelta}^{\hbox{'}}\mathrm{t}+{\alpha}^{\hbox{'}}\cos \left(\omega t\right)+{\upbeta}^{\hbox{'}}\sin \left(\omega t\right)+\cdots +\left(1-{\mathrm{I}}_{\mathrm{t}}\right)\right)\right]+{\in}_{\mathrm{t}} $$

with ∈_t_ = φ_1_∈_t − 1_ + ⋯ +  φ_p_∈_t − p_ + ⋯ +  Z_t_ +  θ_1_ *Z*_t − 1_ + ⋯ +  θ_q_Z_t − q_ ,

where p corresponded to the order of the autoregressive part of the model errors and q indicated the moving average order.

The orders of the components of the ARMA process and the regression coefficients were selected using the Bayesian Information Criterion (BIC) [25]. The standardized residuals of the final model were analysed to verify absence of autocorrelation and partial autocorrelation.

#### Positive WNV cases in raptors confirmed by laboratory tests

To support the plausibility of our evidence, raptors admitted to TRC between 2007 and 2014 that were suspected of West Nile (WN) disease based on clinical signs were assessed by the Veterinary Diagnostic Laboratory of the University of MN (VDL). A raptor admitted to TRC was considered to be suspicious for WN infection if it presented clinical signs typical of the disease in combination with either an elevated white blood cell count (absolute heterophilia) or splenomegaly as demonstrated on radiology. Typical clinical manifestations include neurological signs (including head tilt, nystagmus, and tremors), blindness or visual impairment in Cooper’s hawks, northern goshawks, red-tailed hawks and bald eagles, repetitive head movements (“bobble head”), ataxia, circling, and dysphagia in great horned owls. In hawks (buteos and accipiters), exudative chorioretinal lesions and chorioretinal scarring in a linear pattern were highly suggestive of WN disease. Additionally, other raptors with non-specific signs such as emaciation, dehydration, and dull mentation could be considered as clinical suspicion of WN infection.

During this period different specific studies underwent necropsies in suspicious raptors and performed histopathological analysis. A raptor was considered positive for WNV when individual or pooled tissues (brain, heart, and kidney) were positive for WNV RNA by PCR [26] and/or if WNV antigen was detected in tissue sections of at least one organ by immunohistochemistry using a monoclonal antibody specific for WNV antigen [22].

#### Software

Analyses were implemented in the R software [27], with the “base” and “hts” [19], “forecast” [28], and “nlme” packages [29].

## Results

### Exploratory descriptive analysis

A total of 16,595 raptors from 37 different states were admitted to TRC between 1990 and 2014, although our analysis focused only on admissions from the state of MN (*n* = 13,080; 78.8%). Five of the 28 raptor species admitted accounted for 66% of the admissions: 2360 red-tailed hawks (*Buteo jamaicensis*) (18%); 2148 great horned owls (*Bubo virginianus*) (16.4%); 1538 Cooper’s hawks (*Accipiter cooperii*) (11.8%); 1347 American kestrels (*Falco sparverius*) (10.3%); and 1193 bald eagles (*Haliaeetus leucocephalus*) (9.1%). Most raptors fell into 4 taxonomic groups: 5278 hawks (including buteos and accipiters, 39%); 4272 owls (31%); 1897 falcons (14%); and 1208 eagles (9%). Only 425 (3%) raptors belonged to other groups (Table 1: Summarized table of admitted species).

**Table 1:** Summary of the raptors received at The Raptor Center from Minnesota between 1990 and 2014 detailing: avian group, species, number and percentage

Avian Group	Species	Latin name	No. admissions received at The Raptor Center	Percent
Hawk	red-tailed hawk	*Buteo jamaicensis*	2360	18.0%
	Cooper’s hawk	*Accipiter cooperii*	1538	11.8%
	broad-winged hawk	*Buteo platypterus*	618	4.7%
	sharp-shinned hawk	*Accipiter striatus*	380	2.9%
	red-shouldered hawk	*Buteo lineatus*	145	1.1%
	rough-legged hawk	*Buteo lagopus*	123	0.9%
	goshawk (Northern)	*Accipiter gentilis*	93	0.7%
	Swainson’s hawk	*Buteo swainson*	18	0.1%
	ferruginous hawk	*Buteo regalis*	3	0.0%
Owl	great horned owl	*Bubo virginianus*	2148	16.4%
	barred owl	*Strix varia*	797	6.1%
	saw-whet owl (Northern)	*Aegolius acadicus*	372	2.8%
	screech-owl (Eastern)	*Megascops asio*	368	2.8%
	great gray owl	*Strix nebulosa*	197	1.5%
	long-eared owl	*Asio otus*	136	1.0%
	snowy owl	*Bubo scandiacus*	111	0.8%
	short-eared owl	*Asio flammeus*	92	0.7%
	boreal owl	*Aegolius funereus*	43	0.3%
	hawk-owl (Northern)	*Surnia ulula*	8	0.1%
Falcon	kestrel (American)	*Falco sparverius*	1347	10.3%
	peregrine falcon	*Falco peregrinus*	300	2.3%
	merlin	*Falco columbarius*	244	1.9%
	prairie falcon	*Falco mexicanus*	6	0.0%
Eagle	bald eagle	*Haliaeetus leucocephalus*	1193	9.1%
	golden eagle	*Aquila chrysaetos*	15	0.1%
Others	osprey	*Pandion haliaetus*	225	1.7%
	turkey vulture	*Cathartes aura*	132	1.0%
	harrier (Northern)	*Cyrcus cyaneus*	68	0.5%
			13,080	100.0%

### Time series analysis

The number of admitted raptors increased over time; the median number of admissions per year was 542 with a minimum of 318 in 1990 and a maximum of 818 in 2013. Admissions increased annually in July and August.

When the admissions were analyzed together, considering a unique time series, did not appear to signal the WNV circulation. To distinct underlying patterns and identify a subset of raptors as indicator of WNV circulation, the series had to be grouped by taxomonic group, species, age and clinical signs.

**Fig. 1:**
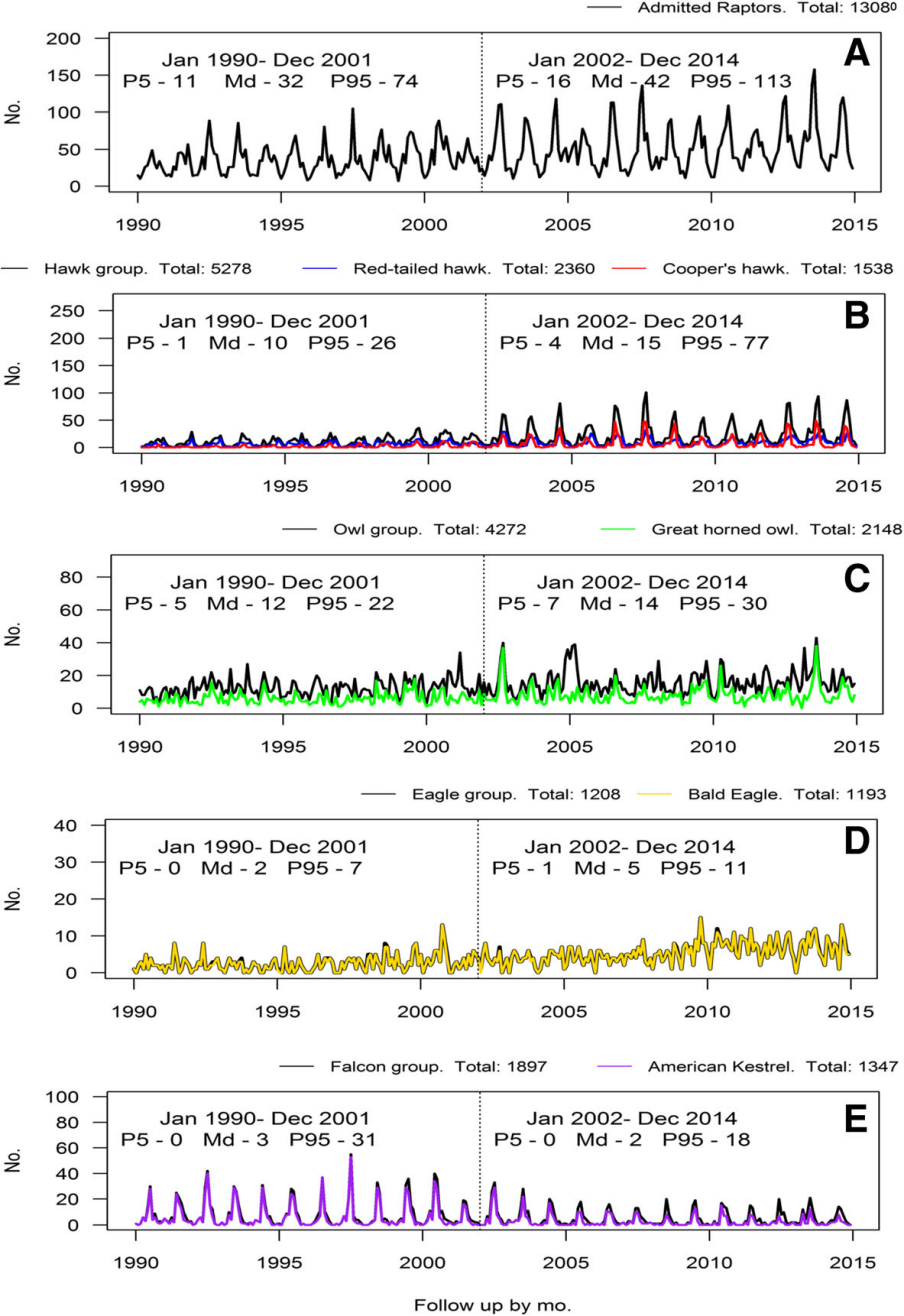
**a**-**e** Time series plots of total admissions, avian groups, and most representative raptors

Exploring the admissions grouped by avian taxonomic group, the results showed that after 2002 the admissions of hawks evidenced a marked increase, whereas in the owl and eagle groups the increases were small. In contrast, the number of admissions of falcons decreased over time. Seasonal patterns were more evident in hawks and falcons than in owls and eagles (see Fig. 1a–e).

ARMA models are used for our data analysis because they are powerful tools that can be directly fitted using standard packages in R. However other families of continuous-time ARMA processes [30], or nonparametric regression models based on P-splines [31] can be explored in further research.

**Fig. 2:**
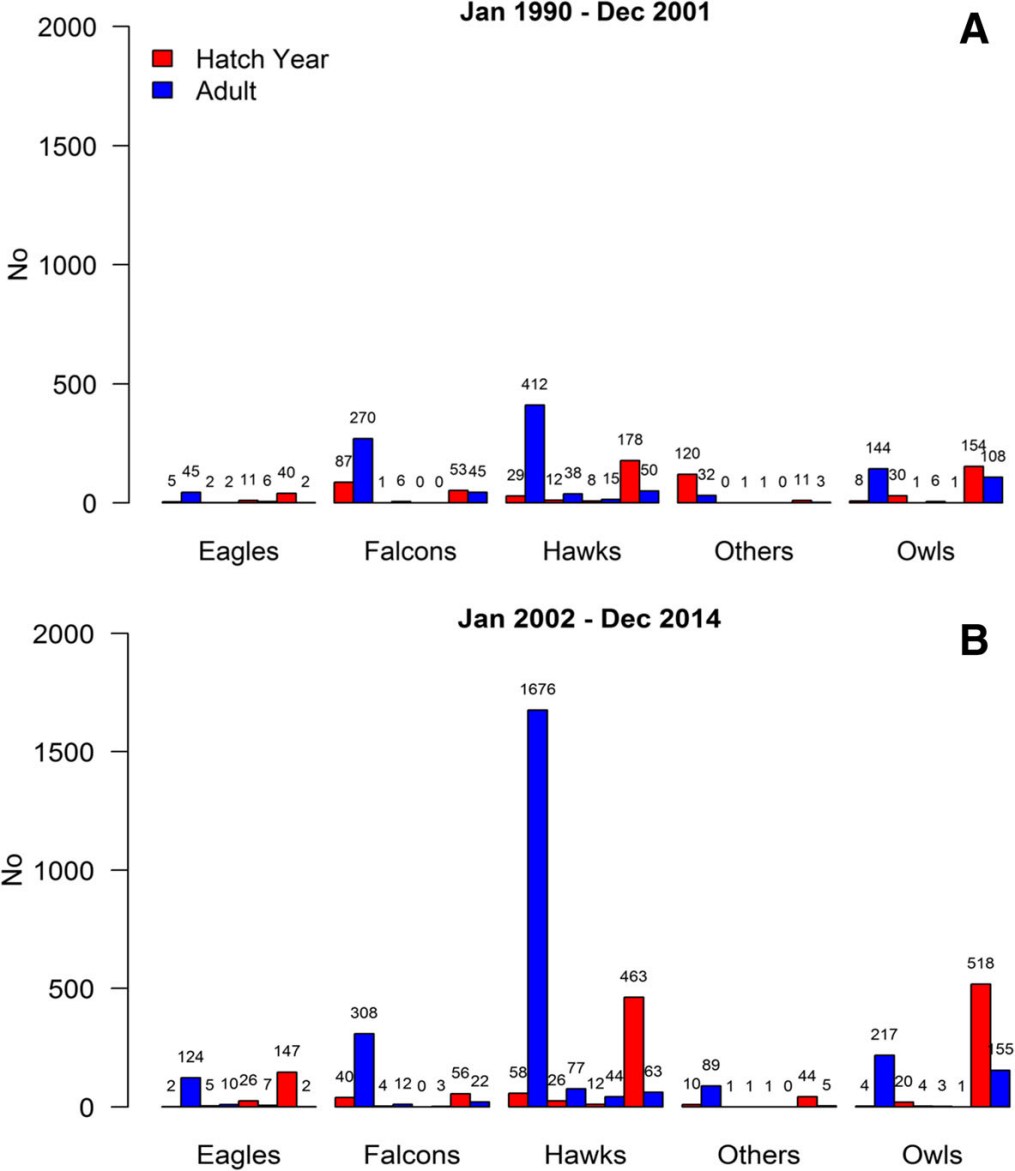
**a**-**b** Frequency of admissions between July–October by avian group and age category before and after 2002

Examining admissions by age, hatch year birds accounted for 33.1% admissions with a marked increase in July and August over all the period. However, it is interesting to varied after the incursion of WNV. Indeed, after 2002 during the periods of WNV circulation (between July–October), the proportions of hatch year in hawks increased, whereas in owls increased the proportion of adults (Fig. 2a-b).

The signs compatible with WNV rose between July and October after its incursion in 2002, whereas the amount of admissions with no problems noted a decrease.

### Identification of raptor subpopulations that potentially indicate WNV circulation

**Fig. 3:**
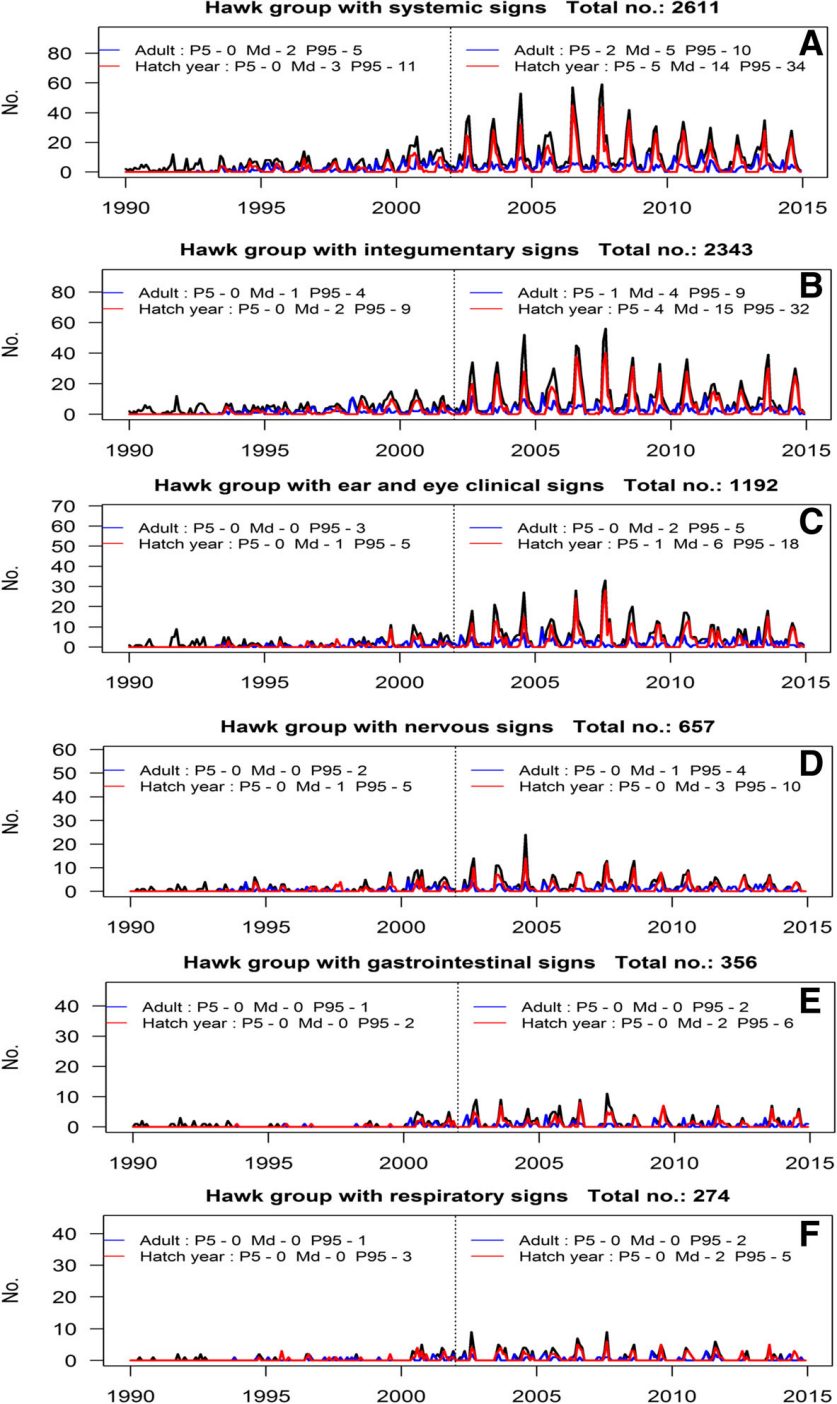
**a**-**f** Time series plots of monthly admissions of hawks according to clinical signs and age

The cojoint analysis of time series considering taxonomic groups, age and clinical signs suggested that the admissions of hawks with compatible clinical signs could indicate the circulation of WNV. The admissions of the hawk group showed the most evident changes before and after 2002 and the monthly medians of systemic, integumentary, ocular problems, ear injury, nervous, gastrointestinal, respiratory signs or other non-specific signs increased signicantly after 2002 (*p*-value < 0.005, Mann-Whitney test) (Fig. 3a–f).

The monthly patterns of clinical signs in this subset were regular before 2002. However, in the summer of 2002 and in consecutive summers, the number of admissions for the most frequent clinical signs significantly increased in comparison with the 1990–2001 period. These increases coincided with the incursion of WNV detected in humans, horses and birds in MN. These changes were more marked in hatch year hawks.

Based on these findings all hawk admissions showing WNV-like signs (namely, systemic, integumentary, ocular problems, ear injuries, nervous, gastrointestinal or non-specific clinical signs) were selected as indicators of WNV circulation.

The changes observed in the patterns during the incursion of WNV were not so apparent in other taxonomic groups.

### Interrupted time series analysis (ITS) in hawks showing WNV-like signs before and after 2002

The results of the ITS model provided statistical evidences about the augment in the number of hawks showing WNV-like signs admittted to TRC during the periods with circulation of WNV. This effect was assessed by examining and comparing the coefficients of the regression model before and after 2002 (see Table 2).

**Table 2:** Coefficients of the regression model and errors fitted to an ARMA structure

Coefficients of the model before 2002	Period January 1990–December 2001				Coefficients of the model after 2002	Period January 2002–December 2014			
	Value Std	Error	*t*-value	*p*-value		Value Std	Error	*t*-value	*p*-value
Intercept	4.04	0.83	4.87	0.00	Intercept	21.50	3.80	5.66	0.00
δ	0.08	0.01	7.66	0.00	δ^′^	−0.004	0.02	−0.22	0.83
*β*_12_	−5.79	0.57	−10.10	0.00	*β*_12_^′^	−16.60	1.05	−15.85	0.00
*α*_12_	−3.20	0.57	−5.60	0.00	*α*_12_^′^	−12.96	1.04	−12.42	0.00
*β*_6_	−0.36	0.54	−0.68	0.50	*β*_6_^′^	8.55	0.98	8.69	0.00
*α*_6_	−2.89	0.54	−5.36	0.00	*α*_6_^′^	−4.96	0.98	−5.05	0.00
*α*_4_	1.08	0.49	2.21	0.03	*α*_4_^′^	6.90	0.89	7.72	0.03
*α*_3_	−1.58	0.44	−3.62	0.00	*α*_3_^′^	−4.53	0.80	−5.69	0.00
ϵ_t_	Z_t_ + 0.22_1_Z_t − 1_								
AIC: 1913.46 BIC: 1983.83 logLik −937.73									
Standardized residuals: Min: −3.47 Q1: −0.61 Med: −0.12 Q3: 0.47 Max: 4.60									

**Fig. 4:**
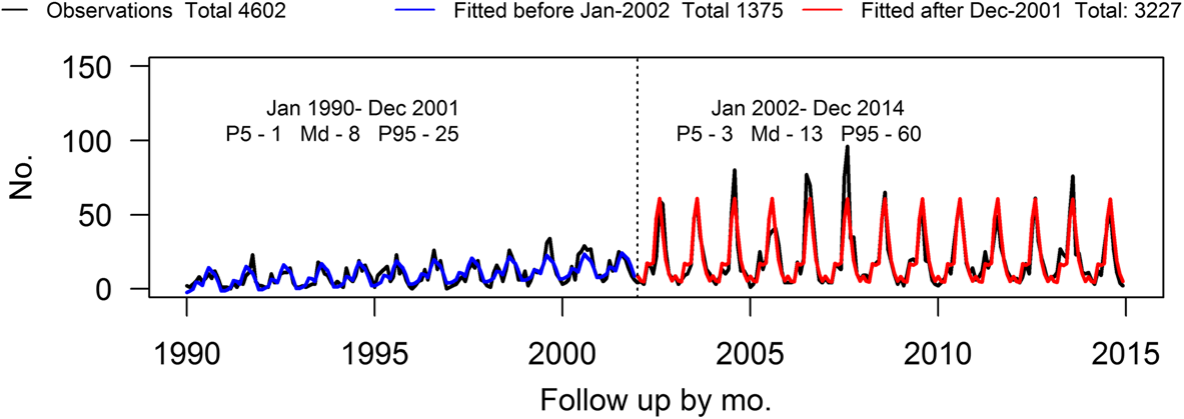
Time series of hawks admitted with clinical signs compatible with West Nile and fitted values

The coefficients of the trigometric covariates (β_i_ and α_i_ for *i* = 12, 6, 4 and 3) showed that the frequency of admissions of hawks with signs compatible with WNV followed a marked annual seasonality and a less evident cyclical pattern every 6, 4 and 3 months (Fig. 4 and Table 2). The value of δ evidenced that the overall trend of admissions increased between 1990 and 2002. In contrast, the trend after 2002 represented by δ’ was null over time.

The circulation of WNV appeared to increase the frequency of hawks showing WNV-like signs almost 3 times during July and August. Furthermore, the high standard deviations obtained in the errors after 2002 indicated that the circulation of WNV also increased the uncertainty in the number of admissions over time.

The diagnostic checking evidenced the lack of autocorrelation or partial autocorrelation in the residuals of our model.

### WNV cases in raptors confirmed by laboratorial tests

Between 2007 and 2014 a total of 333 raptors were submitted by TRC to the Minnesota Veterinary Diagnostic Laboratory (VDL) as suspicious for WNV; 162 were WNV positive. Most cases (110, 67.9%) were hawks (i.e. red-tailed hawks, Cooper’s hawks and northern goshawks), and most positive hawks (80%) were hatch year birds. The most frequent clinical signs observed in these raptors were systemic disorders (i.e. dehydratation, depression, weakness, emaciation), neurological (i.e. head tremors, postural problems, imbalance, head tilts) and ocular signs; followed by integumentary (i.e. soft tissue injuries, damaged feathers/cere/ft or bumblefoot) and gastrointestinal signs such as anorexia, and lesions in mouth, among others.

## Discussion

Our results demonstrate that raptor rehabilitation data, specifically hawks, shows marked temporal differences before and after the incursion of WNV in MN. These results coincide with previous studies conducted in different regions of USA, in which these species showed to be highly susceptible to WNV [7, 32]. The conclusion that these changes were likely due to WNV were further supported by the abrupt change in the frequency of admissions of hawks with clinical signs consistent with WNV in 2002, and the diagnostic confirmation of WNV-positive cases in hawks post-2002. This study serves as a proof-of-concept that some animal health events, such as the incursion of a new infectious disease, result in aberrations of long-term data trends collected by rehabilitation centers. Thus, near real-time analysis of rehabilitation data within a syndromic surveillance framework could potentially contribute to the detection of health anomalies and monitoring of animal health trends in wildlife populations.

Various WNV surveillance programs have been implemented in humans, horses, birds and mosquitoes throughout the United States, including MN, though many programs relying on active surveillance for WNV have been discontinued due to low sensitivity and high cost [33]. Thus, the syndromic surveillance approach here demonstrates the potential impact that an alternative and affordable source of information may have in supporting early detection and monitoring of WNV. Ultimately, the working example here shows the potential of data from wildlife rehabilitation centers to signal the introduction and circulation of emergent pathogens in wild animal populations.

Additionally, because raptors are placed at the top of the food chain and occupy broad areas, monitoring of raptor admissions may help to monitor the health status of other populations in the ecosystem [34, 35]. Routine monitoring of rehabilitation center data may help to assess the impact and evolution of a specific disease on wildlife and provide a better understanding of its transmission in the natural ecosystems. However, monitoring of raptors admissions may not be straightforward. For example, when raptor admissions were analyzed collectively, there was no evident change in patterns that could signal WNV circulation. In contrast, when frequency of admissions was assessed by taxonomic group, age and clinical signs, it was evident that certain patterns changed substantially coinciding with the occurrence of WNV outbreaks. Temporal variations were clear in hawks with WNV-like signs during the summer of 2002 and consecutive summers, especially in hatch year birds. Thus, syndromic surveillance programs based on rehabiliaton data will need to not only monitor overall trends, but also focus on identifying aberrations or trends when data is subset by taxomony, age, and suites of clinical signs. Monitoring various taxomonic or syndromic subsets for data aberrations is particularly important if syndromic surveillance is meant to detect the emergence of pathogens in a geographic region rather than monitor pathogens already circulating.

Despite the potential of wildlife rehabilitation center data for syndromic surveillance, there are certain limitations that should be considered. Most importantly, monitoring of those data is only a proxy for wildlife health status, and consequently, other complementary information might be essential to support evidence before extracting definitive conclusions. Additionally, animals admitted into the rehabilitation centers may not be representative of the status of the wildlife population because animal collection is linked to the human activity. Moreover, the timeliness of these collections can also be determined by the degree of awareness. Presence of such collection bias may have, for example, affected our results given that the number of raptors found after 2002 was higher. However, the total number of admissions did not significantly change (Fig. 1a–e) as much as the specific pattern of young hawk admissions with WNV-like signs (Fig. 3a–f).

Another potential limitation may be due to the lack of specificity of clinical signs. Different pathogens and conditions may result in similar clinical patterns. For that reason, assessment of underlying patterns by species, age or clinical signs complemented with specific diagnosis in the event of peaks in admissions might be necessary to identify indicators for specific diseases. Given that these limitations are acknowledged and, ideally, controlled, wildlife rehabilitation center data may serve as an affordable and reliable source of information for monitoring conditions at the interface of public, animal, and environmental health, thus supporting the One Health concept at local and regional levels.

## Conclusions

Retrospective analysis of raptor rehabilitation data evidenced marked temporal differences before and after the incursion of West Nile Virus in MN, indicating that monitoring of data routinely collected by wildlife rehabilitation centers has the potential to capture the geographical emergence of a new pathogen in free-ranging wildlife. This study serves as a proof-of-concept that aberrations in long-term trends in datasets collected by wildlife rehabilitation centers may reflect animal health events occurring within free-ranging wildlife populations. These results demonstrate that wildlife rehabilitation centers may serve as an affordable resource to complement the routine monitoring, and ultimately, early detection and prevention of public, animal, and environmental health conditions in the country.

## Abbreviations

AIC: Akaike Information Criterion
AIV: Avian Influenza Virus
ARMA: AutoRegressive-Moving Average
BIC: Bayesian Information Criterion
ITS: Interrupted Time Series
MN: Minnesota
PCR: Polymerase Chain Reaction
RNA: Ribonucleic Acid
TRC: The Raptor Center
USA: United States of America
VDL: Veterinary Diagnostic Laboratory
WNV: West Nile Virus

## References

[1] Butler D (2006). Disease surveillance needs a revolution. Nature.

[2] Jebara KB (2004). Surveillance, detection and response: managing emerging diseases at national and international levels. Rev Sci Tech Off Int Epiz.

[3] Morner T, Obendorf DL, Artois M, Woodford MH (2002). Surveillance and monitoring of wildlife diseases. Rev Sci Tech Off Int Epiz.

[4] Thiermann AB (2005). Globalization, international trade and animal health: the new roles of OIE. Prev Vet Med.

[5] Willette M, Ponder J, McRuer DL, Clark EE, Aguirre A (2013). Wildlife Health Monitoring Systems in North America: From Sentinel Species to Public Policy. Conservation Medicine: Applied Cases of Ecological Health.

[6] Randall NJ, Blitvich BJ, Blanchong JA (2012). Efficacy of wildlife rehabilitation centers in surveillance and monitoring of pathogen activity: a case study with West Nile virus. J Wildl Dis.

[7] Henning KJ (2004). What is syndromic surveillance?. MMWR Morb Mortal Wkly Rep.

[8] Katz R, May L, Baker J, Test E (2011). Redefining syndromic surveillance. J Epidemiol Glob Health.

[9] Camacho M, Hernández JM, Lima-Barbero JF, Höfle U (2016). Use of wildlife rehabilitation centres in pathogen surveillance: a case study in white storks (Ciconia Ciconia). Prev Vet Med.

[10] Cox-Witton K, Reiss A, Woods R, Grillo V, Baker RT, Blyde DJ, Boardman W, Cutter S, Lacasse C, McCracken H, Pyne M (2014). Emerging infectious diseases in free-ranging wildlife–Australian zoo based wildlife hospitals contribute to national surveillance. PLoS One.

[11] Doell D, Locky DA (2016). Trends in wildlife intake at a rehabilitation center in Central Alberta: a retrospective analysis of birds, mammals, and herptiles, from 1990 through 2012. J Wildlife Rehabil.

[12] Pultorak E, Nadler Y, Travis D, Glaser A, McNamara T, Mehta SD (2011). Zoological institution participation in a West Nile virus surveillance system: implications for public health. J Public Health.

[13] Stitt T, Mountifield J, Stephen C (2007). Opportunities and obstacles to collecting wildlife disease data for public health purposes: results of a pilot study on Vancouver Island. British Columbia Can Vet J.

[14] Beasley DW, Barrett AD, Tesh RB (2013). Resurgence of West Nile neurologic disease in the United States in 2012: what happened? What needs to be done?. Antivir Res.

[15] Bell JA, Brewer CM, Mickelson NJ, Garman GW, Vaughan JA (2005). West Nile virus Epizootiology, central Red River valley, North Dakota and Minnesota, 2002-2005. Emerg Infect Dis.

[16] Nemeth N, Kratz G, Edwards E, Scherpelz J, Bowen R, Komar N (2007). Surveillance for West Nile virus in clinic-admitted raptors, Colorado. Emerg Infect Dis.

[17] Athanasopoulos G, Ahmed RA, Hyndman RJ (2009). Hierarchical forecasts for Australian domestic tourism. Int J Forecast.

[18] Hyndman RJ, Ahmed RA, Athanasopoulos G, Shang HL (2011). Optimal combination forecasts for hierarchical time series. Comput Stat Data Anal.

[19] Hyndman RJ, Lee A, Wang E, Wickramasuriya S, Wang ME. Package ‘hts’. Hierarchical and Grouped Time Series. R package version 4.5. 2015. http://CRAN.R-project.org/package=hts. Accessed 21 Jun 2017.

[20] Mann HB, Whitney DR. On a test of whether one of two random variables is stochastically larger than the other. Ann Math Stat. 1947:1950–60.

[21] Ellis AE, Mead DG, Allison AB, Stallknecht DE, Howerth EW (2007). Pathology and epidemiology of natural West Nile viral infection of raptors in Georgia. J Wildl Dis.

[22] Wünschmann A, Shivers J, Bender J, Carroll L, Fuller S, Saggese M, van Wettere A, Redig P (2005). Pathologic and immunohistochemical findings in goshawks (Accipiter Gentilis) and great horned owls (Bubo Virginianus) naturally infected with West Nile virus. Avian Dis.

[23] Afonso ET, Minamisava R, Bierrenbach AL, Escalante JJ, Alencar AP, Domingues CM, Morais-Neto OL, Toscano CM, Andrade AL (2013). Effect of 10-valent pneumococcal vaccine on pneumonia among children. Brazil Emerg Infect Dis.

[24] McDowall D. Interrupted time series analysis, Vol. 21. Sage. 1980.

[25] Schwarz G (1978). Estimating the dimension of a model. Ann Stat.

[26] Lanciotti RS, Kerst AJ, Nasci RS, Godsey MS, Mitchell CJ, Savage HM, Komar N, Panella NA, Allen BC, Volpe KE, Davis BS (2000). Rapid detection of West Nile virus from human clinical specimens, field-collected mosquitoes, and avian samples by a TaqMan reverse transcriptase-PCR assay. J Clin Microbiol.

[27] Team RC. R: A Language and Environment for Statistical Computing. Vienna: R Foundation for Statistical Computing. 2014. http://www.R-project.org.

[28] Hyndman RJ, O'Hara-Wild M, Bergmeir C, Razbash S, Wang E, Hyndman MR. Package ‘forecast’. 2007. http://www.cran.r-project.org/web/packages/forecast/forecast.pdf. Accessed 23 Feb 2017.

[29] Pinheiro J, Bates D, DebRoy S, Sarkar D. R Core Team. nlme: Linear and Nonlinear Mixed Effects Models. R package version 3.1–127. 2016. http://CRAN.R-project.org/package=nlme. Accessed 6 Feb 2017.

[30] Arratia A, Cabana A, Cabana EM (2016). A construction of continuous-time ARMA models by iterations of Ornstein-Uhlenbeck processes. SORT-Statistics and Operations Research Transactions.

[31] Eilers PHC, Marx BD, Durban M (2015). Twenty years of P-splines. SORT-Statistics and Operations Research Transactions.

[32] Kilpatrick AM, LaDeau SL, Marra PP (2007). Ecology of West Nile virus transmission and its impact on birds in the western hemisphere. Auk.

[33] Hadler JL, Patel D, Nasci RS, Petersen LR, Hughes JM, Bradley K, Etkind P, Kan L, Engel J (2015). Assessment of arbovirus surveillance 13 years after introduction of west Nile virus, United States. Emerg Infect Dis.

[34] Bowerman WW, Roe AS, Gilbertson MJ, Best DA, Sikarskie JG, Mitchell RS, Summer CL (2002). Using bald eagles to indicate the health of the Great Lakes' environment. Lake Reserv. Manage.

[35] Giesy JP, Bowerman WW, Mora MA, Verbrugge DA, Othoudt RA, Newsted JL, Summer CL, Aulerich RJ, Bursian SJ, Ludwig JP, Dawson GA (1995). Contaminants in fishes from Great Lakes-influenced sections and above dams of three Michigan rivers: III. Implications for health of bald eagles. Arch Environ Contam Toxicol.

